# Fully automatic segmentation of short and long axis cine cardiac MR

**DOI:** 10.1186/1532-429X-11-S1-P226

**Published:** 2009-01-28

**Authors:** Maxim Fradkin, Cybele Ciofolo-Veit, Benoit Mory, Gilion Hautvast, Marcel Breeuwer

**Affiliations:** 1 Philips Healthcare, Suresnes, France; 2 Philips Healthcare, Best, Netherlands

**Keywords:** Left Ventricle, Automatic Segmentation, Segmented Contour, Left Ventricle Volume, Left Ventricle Myocardium

## Introduction

Quantitative analysis of cardiac function requires delineation of the left ventricle (LV) in cine cardiac MR (CMR). Typically, this is done using short-axis (SA) images, however, acquisition of several long-axis (LA) views has become quite common. The latter can be used for the accurate and reproducible determination of the basal SA slice, known as one of the major inter-observer variability factors in SA LV measurements [1]. Since manual LV delineation is very tedious and time-consuming, automatic segmentation methods, enabling to obtain reproducible LV measurements, are highly desirable.

## Purpose

We propose a fully automatic method for delineation of the endo- and epicardial contours in SA and LA cine CMR images in order to provide automatic, accurate quantitative left-ventricular functional assessment.

## Methods

**Figure 1:**
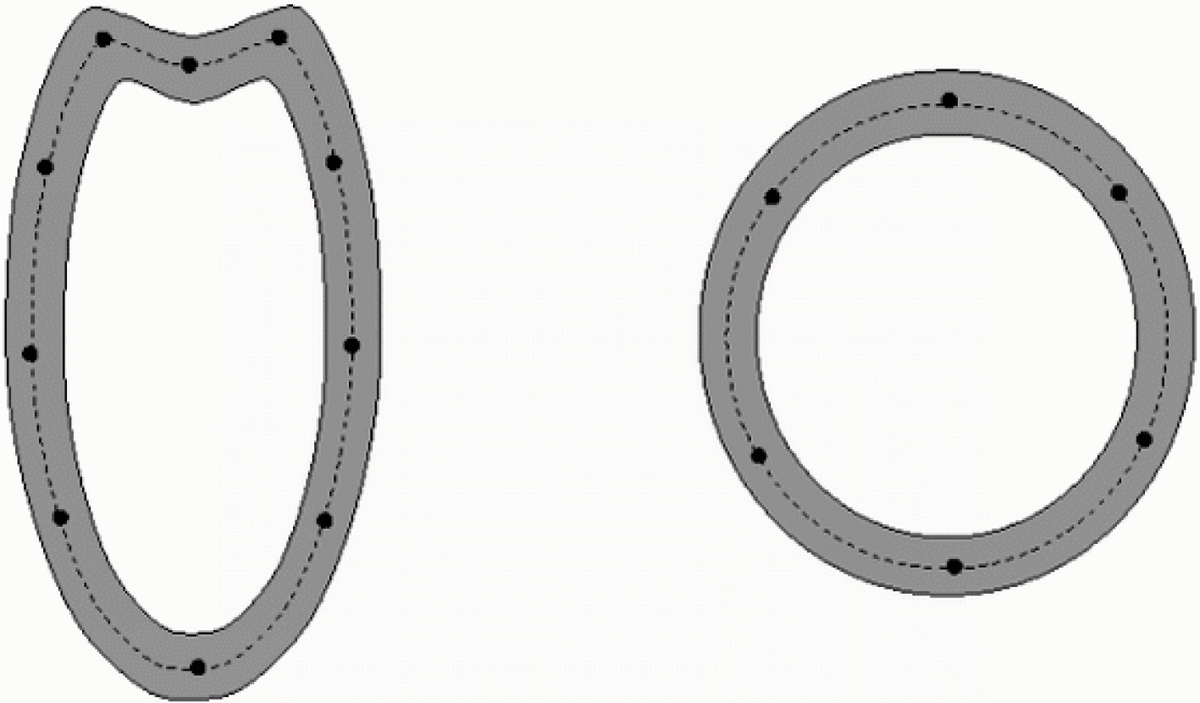
**Deformable templates modeling LA** ***(left)*** **and SA** ***(right)*** **: centerline** ***(dashed)*** **is defined by a few nodes, the template width (grey ribbon) may vary from node to node**.

Our segmentation workflow consists of two steps: (1) automatic detection and delineation of the LV myocardium and valve plane in LA image(s); (2) segmentation of SA myocardial contours, using the results of step 1. For modelling the LV, we use ribbon-like template, relying on interpolating splines and defined by a very few nodes; its shape is controlled by nodes' position and the template's width at each node (Figure 1). The template is deformed using *greedy optimization* framework [2]. The optimization criterion comprises three terms, responsible for templates' shape smoothness, border contrast and region homogeneity. The optimization is done in coarse-to-fine manner, by relaxing the deformation type (from rigid to local) and adjusting the influence (weights) of different terms.

The initial position of the LA template is derived from the image acquisition geometry, while that for the SA templates is obtained by projecting the LA segmented contours on the corresponding SA slices.

We quantitatively assessed the performance of the method on a database of 35 cine CMR acquisitions (Philips Gyroscan NT Intera 1.5 T, SSFP protocol, TE = 1.6 ms, TR = 3.3 ms, flip angle 50° for SA and TE = 1.5–1.7 ms, TR = 3.0–3.4 ms, flip angle 55° for LA). The SA ones included 9 to 14 slices (325 slices in total), and the LA ones 1 to 2 views (40 views in total). The assessment was made by comparing the automatically segmented contours with Gold Standard manual delineations provided by an expert.

## Results

**Figure 2:**
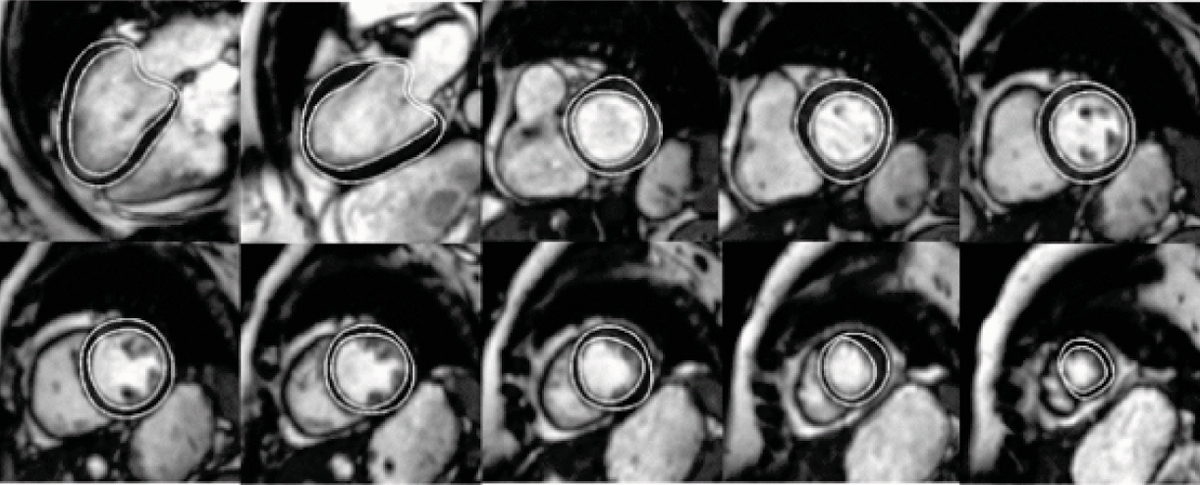
**LV segmentation example for one patient:** ***(from left to right, top to bottom)*** **: LA 4 chambers and 2 chambers; consecutive slices of SA volume from valve to apex**.

For SA images, the mean positioning error (MPS) was 1.3 ± 0.5 mm for the endocardium and 1.5 ± 0.8 mm for the epicardium, for a pixel size of 1.4–1.8 mm. For LA images, the MPS was 1.3 ± 0.4 mm (endocardium) and 1.1 ± 0.4 mm (epicardium), for a pixel size of 1.3–1.8 mm. Both results are under one pixel accuracy and comparable with inter- and intra-observer variability. An excellent correlation was found between "manual" and "automatic" LV volumes (r = 0.98, P < 0.001), with mean difference 2.5 ± 7.6 ml (5.5 ± 3.9%). Note that all results were obtained without any user interaction. An example of segmentation results for one patient (8 SA slices and 2 LA views) is shown in Figure 2. We validated the method for the end diastolic (ED) phase only, since the segmentation of the whole cardiac cycle can be then obtained using automatic propagation of ED contours [3]. In earlier investigations, we already showed that propagation preserves the segmentation accuracy within acceptable ranges while being much faster than phase-by-phase segmentation.

## Conclusion

We presented a robust, accurate and efficient method for the fully automatic delineation of the myocardium contours in LA + SA cine CMR images, which can be used for accurate LV functional assessment.
